# Organizing the dissemination and implementation field: who are we, what are we doing, and how should we do it?

**DOI:** 10.1186/s43058-024-00572-1

**Published:** 2024-04-11

**Authors:** Gretchen J. R. Buchanan, Lindsey M. Filiatreau, Julia E. Moore

**Affiliations:** 1 Department of Family Medicine and Community Health, Hennepin Healthcare Research Institute, Minneapolis, MN and University of Minnesota Medical School, MN Minneapolis, USA; 2 Division of Infectious Diseases, School of Medicine, Washington University in St. Louis, MO St. Louis, USA; 3 The Center for Implementation, ON Toronto, Canada

**Keywords:** Dissemination and implementation, Implementation science, Translational science, Translational science spectrum, Translational research continuum, Evidence to practice gap, Implementation support practitioners

## Abstract

Two decades into its tenure as a field, dissemination and implementation (D&I) scientists have begun a process of self-reflection, illuminating a missed opportunity to bridge the gap between research and practice—one of the field’s foundational objectives. In this paper, we, the authors, assert the research-to-practice gap has persisted, in part due to an inadequate characterization of roles, functions, and processes within D&I. We aim to address this issue, and the rising tension between D&I researchers and practitioners, by proposing a community-centered path forward that is grounded in equity.

We identify key players within the field and characterize their unique roles using the translational science spectrum, a model originally developed in the biomedical sciences to help streamline the research-to-practice process, as a guide. We argue that the full translational science spectrum, from basic science research, or “T0,” to translation to community, or “T4,” readily applies *within* D&I and that in using this framework to clarify roles, functions, and processes within the field, we can facilitate greater collaboration and respect across the entire D&I research-to-practice continuum. We also highlight distinct opportunities (e.g., changes to D&I scientific conference structures) to increase regular communication and engagement between individuals whose work sits at different points along the D&I translational science spectrum that can accelerate our efforts to close the research-to-practice gap and achieve the field’s foundational objectives.

Contributions to the literature

- Providing clarity regarding the distinct groups of individuals involved in D&I science and practice from researchers to the communities impacted by the change and outline key roles of these unique sets of actors.
- Specifying the range of activities, from theoretical research to applied implementation, involved in D&I science and practice using a translational structure.
- Identifying existing gaps (e.g., poor integration of research into existing implementation efforts) that impede attainment of the shared vision of D&I science and practice and propose solutions to these gaps.

## Introduction

Though still in its infancy, the field of dissemination and implementation science (D&I) [1, 2] is facing challenges related to the growing gap between the science and practice of implementation [1, 3, 4]. D&I is the scientific study of translating research findings and evidence-based interventions into everyday practice; in the current state of the D&I literature, this often means that a practice developed by one group of actors is being implemented into the everyday practice of others [5]. A premortem by Beidas and colleagues [4] highlighted several factors stagnating the field, including closure of the evidence-to-practice gap [6–9], insufficient impact, and inability to align timelines and priorities with partners [1]. This commentary aims to establish further clarity regarding who “we” are as a field, what we are doing, and how we can collectively work to achieve shared goals of improved population health in D&I. This refers to the collective “we” of those engaged in D&I work.

In clarifying key components of D&I, important lessons can be drawn from more established fields. For example, when reflecting on disciplines such as mathematics and physics, one notes the emergence of two broad areas of scholarship—theoretical and applied—within these fields. These scholarship areas fill distinct, but important roles within their fields. Here, the authors posit that D&I science could be similarly broken down into theoretical and applied scholarships. In this paper, we, the authors, elaborate on the functions of these differential scholarships, and the functions of professionals working in the large and ever-growing field of implementation practice.

While many have noted D&I aims “to promote the adoption and integration of evidence-based practices, interventions, and policies into routine health care and public health settings to improve the impact on population health,” [10] specificity in how to achieve this outcome has been elusive. In this article, we propose that the field must first define the actors and audiences across the implementation spectrum and how each group connects with others. Subsequently, the field can strengthen the infrastructures that facilitate these connections. In this article, we aim to address the rising tension between implementation scientists, implementation support practitioners, delivery systems [11], and communities by proposing a path forward that is community-oriented and grounded in equity, thereby upholding every actor’s place at the D&I table. We draw on principles well-established in the field of translational science to better align D&I towards both improved ideas and real-world impact. We note that our mental model as authors is that success for D&I would be defined as impact at the community or population levels. We recognize this is not the mental model held by all people working in D&I, but believe even for those whose focus is not on population impact, we can collectively work together to achieve these outcomes and impact practice [12].

## Who are we?

To date, much of the discussion around the direction of D&I has been researcher-centric [13]. To promote greater equity within the discipline (i.e., to reduce disparities in whose voices are heard within the field of D&I), we would like to expand the existing discourse to include the entire spectrum of professionals who work in implementation, including communities, delivery systems, implementation support practitioners, intermediaries, non-implementation science researchers (e.g., interventionists), and applied and theoretical D&I researchers. Including the entire implementation workforce in a description of the field provides opportunities to see where practitioners have not been empowered to exert influence and to change these inequities. While D&I professionals are likely to fill more than one role at a time or during their careers and may hold perspectives that are therefore representative of a number of these D&I actors, we would like to re-center the current conversation within D&I around implementation support practitioners and delivery systems specifically to uphold our commitment to those most directly affected by D&I efforts.

### Communities and individuals impacted by the change

Communities and the individuals who comprise them play a critical role in the success or failure of efforts to implement evidence-based or informed programs and practices (EBPs) within a particular setting [14–17]. Aligned with this principle, there has been a shifting focus from using community-based to community-led research methods across academic disciplines [18, 19]. Funding agencies have also begun to recognize the need for greater community involvement in research, with current directives to engage community partners *across*the research spectrum [20]. As suggested by others, strengthening relationships between communities and individuals working at all levels of implementation should remain a priority in closing the evidence-to-practice gap and upholding equity in D;I; indeed, it is essential [21].

### Practitioners—implementation support practitioners and delivery systems

Implementation has been happening for the entirety of human history. While several scientific fields (e.g., political science, medicine) began formally investigating processes of D&I in the mid-to-late twentieth century—thereby laying the foundation for current research in this area— the distinct field of D&I only emerged in the past few decades, prompted by repeatedly observed barriers to the successful implementation of EBPs [5, 22].

**Fig. 1:**
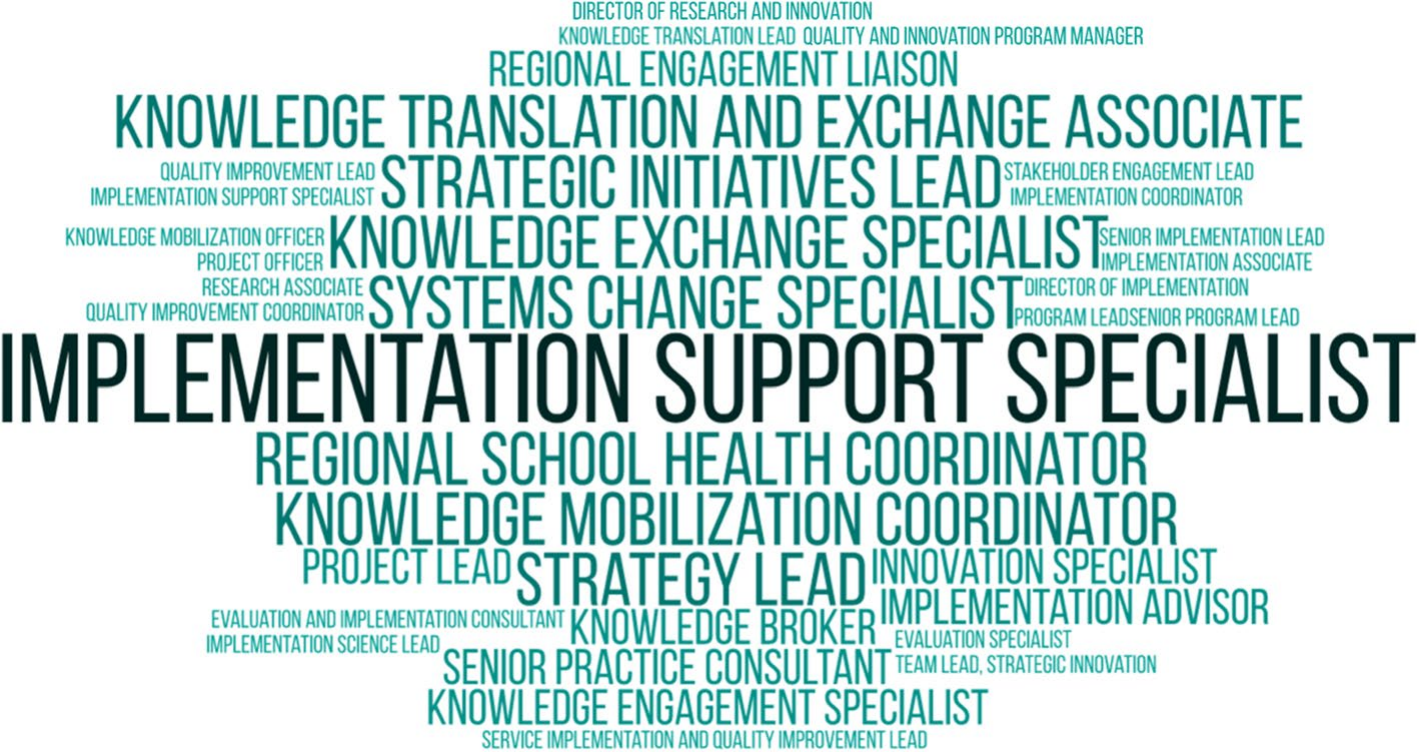
Professional job titles of individuals working directly in implementation or implementation support as identified through the Center for Implementation (In preparation for an event about the roles of implementation support practitioners, an open call was sent out to members of an online community of professionals supporting implementation. People were asked for their current or previous job titles that included an implementation component.)

“Implementation practitioners” are professionals comprised of two distinct groups: implementation support practitioners [23, 24] (e.g., administrators, policy-makers) are involved in planning, engagement, co-creation, strategy selection, capacity building, monitoring, and evaluation; delivery systems (e.g., front-line managers at organizations implementing an EBP) are responsible for implementing the actual practices with professionals, organizations, and the public [11]. Identifying professionals engaged in implementation practice can be difficult as there is inconsistency and terminology; for example, there are over 30 job titles associated with implementation support practitioner roles (see Fig. 1). “Delivery systems” are often unaware of the D&I field or their role as end-users. Implementation researchers appropriately identifying and connecting with delivery systems and implementation support practitioners is key to closing the evidence-to-practice gap and improving impact [4].

### Intermediaries

Globally, there are several intermediary organizations serving to translate findings from D&I to support the implementation of EBPs by delivery systems and implementation support practitioners (e.g., the Collaborative for Implementation Practice; Center for Evidence and Implementation in Australia; Impact Center at the University of North Carolina; Center for Effective Services in Ireland; the Nigerian Implementation Science Alliance). These organizations employ implementation support practitioners and bridge the implementation research-to-practice divide by providing training in implementation-related skills and creating tools to support the selection of appropriate implementation strategies. For example, one intermediary has a mini-course providing an introduction to implementation that has enrolled over 10,000 individuals. Millions of research, government, and philanthropic dollars are being invested in these organizations [25–28]. As implementation researchers and intermediaries, the authors regularly hear from organizations, communities, and individuals that they struggle to access supports in implementation science to address their needs in implementing evidence The demand for this type of work often outpaces the supply, and researchers and funders alike state a clear need for additional resources linking implementation science and practice [29–33].

### Researchers

To better clarify the full spectrum of implementation researchers, researchers whose work is primarily centered on the advancement of implementation ideas (e.g., theory, methods, or framework (TMF) development) are referred to as *theoretical implementation scientists* and those whose work is primarily centered on the direct use of implementation concepts as a method to achieve better clinical or programmatic outcomes as *applied implementation scientists*. Scientists may work on both theoretical and applied projects but tend to focus their programs of research in one or the other and may even identify as one or the other.

Non-D&I researchers are also becoming increasingly interested in D&I, as evidenced by the growing number of D&I training institutes globally (e.g., HIV, Infectious Disease and Global Health Implementation Research Institute (HIGH IRI); University College Cork Implementation Science Training Institute; University of Nairobi Implementation Science Fellowship; Training Institute for Dissemination and Implementation Research in Health (TIDIRH)) [34]. Non-D&I researchers are individuals from distinct substantive areas (e.g., HIV, cancer prevention) who are interested in applying D&I to their work but have limited training in this area. These researchers often aim to draw from the TMFs and evidence from D&I to design, implement, and scale EBPs. They may benefit from increased collaboration with individuals who have worked more squarely in D&I.

## What are we doing?

We, the paper’s authors, entered the field of D&I with the goal of bridging the research-to-practice gap to better improve the lives of people in our areas of scholarship (HIV, mental health). Yet, we have found that our substantively distinct bodies of applied D&I research have unfolded in such a way that we are all currently involved in a range of theoretical implementation research. This journey has not been without difficulty—the further we moved from our applied work and what grounded our science, the less impact we felt we were having. While we found theoretical research important, we felt as though our roles and functions within D&I were less clear. This lack of clarity in our professional self-concept ultimately helped us identify that D&I is not monolithic. Through conversation, we found that articulating the spectrum of theoretical to applied D&I helped us regain the clarity we needed to continue advancing our science. We believe these realizations could also be beneficial to other D&I professionals.

### Leveraging translational science to find clarity

**Fig. 2:**
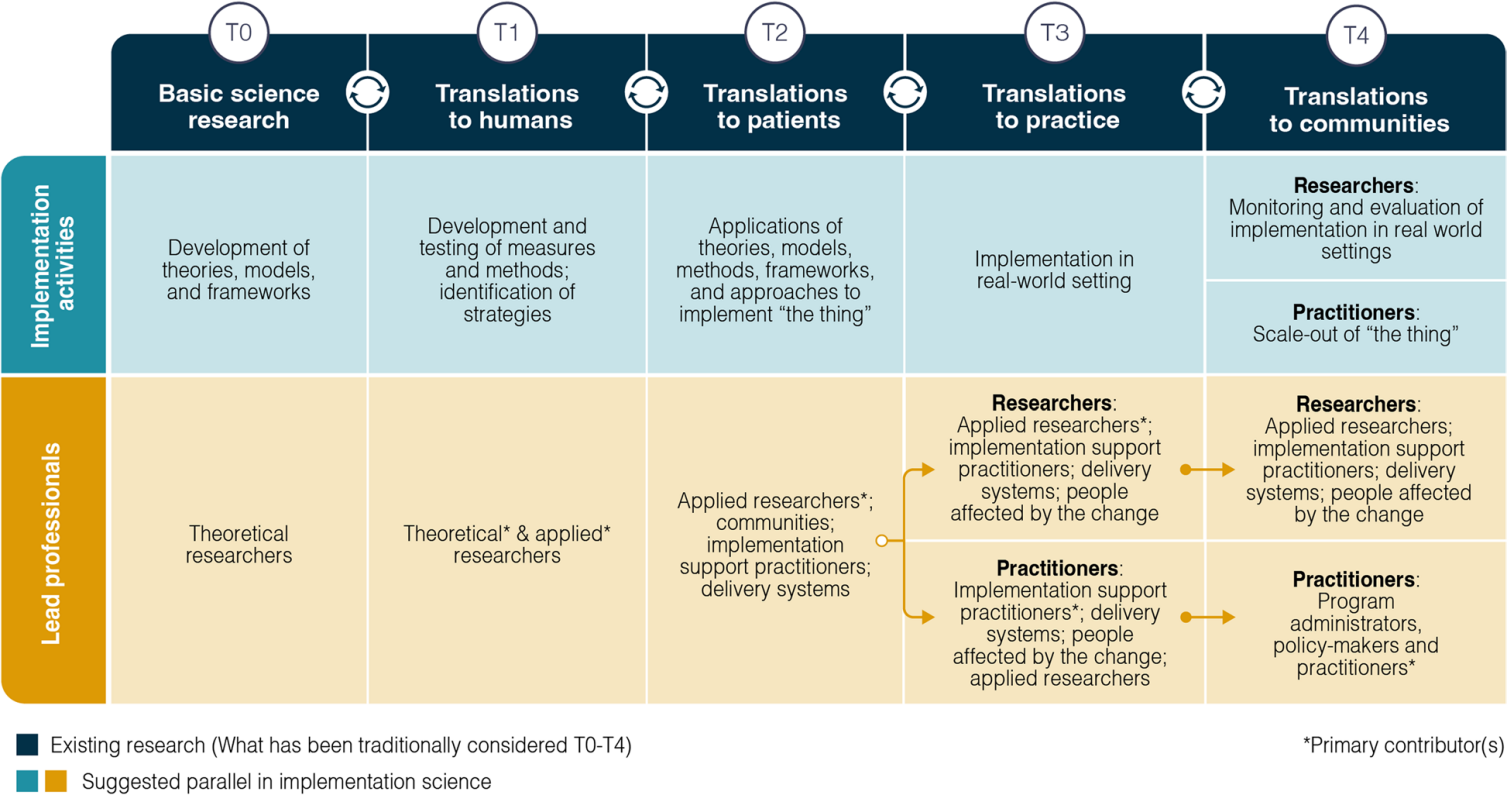
The translational spectrum applied to implementation science

There is extensive literature on moving research findings into practice [35], but the translation of D&I knowledge into practice has received much less attention [1]. Moreover, there is insufficient understanding of which actors are involved at which stages along this spectrum, how each stage contributes to the field, and how these stages, and actors at each of these stages, can connect and achieve shared goals. In Fig. 2, the authors draw on the translational spectrum to address these limitations. The traditional translational spectrum aims to streamline the “bench to bedside” approach and defines the continuum of basic science (stage T0) to public health science (stage T4) [36]. D&I science has long been placed in the T3–T4 segments of the traditional translational spectrum [36]. However, we argue that the full translational spectrum, from T0 through T4, is applicable to D&I. This distinction is often at the core of the tension observed within the field and where our personal struggles in our shifting identities and relationship with D&I research emerged.

In the traditional translational spectrum, T0, “pre-clinical research,” includes bench science and aims to define mechanisms, targets, and strategies for intervention on a general level. In D&I, *theoretical implementation scientists*work on the development of TMFs, and elicitation, description, and modeling of mechanisms. Many of the foundational papers that guide implementation research to date stem from work at this stage [37–43]. T1, “translation to humans,” includes Stage 1 clinical trials and proof of concept science and aims to develop new methods of diagnosis, treatment, and prevention in highly controlled settings. In D&I, *theoretical and applied implementation researchers* focus on translating theoretical constructs (i.e., TMFs) to actual people and developing methods to test these constructs. Examples of this type of research include measurement of implementation domains such as context (e.g., the Organizational Readiness for Change measure) [44] and implementation outcomes (e.g., the NoMAD measure from Normalization Process Theory) [45]. T2, “translation to patients,” includes Stages 2–3 clinical trials and aims to develop clinical applications and evidence-based guidelines for a given disease. In D&I, *applied implementation researchers*focus on identifying implementation constructs relevant to a specific situation, intervention, context, or population where the researchers aim to understand how best to implement. Traditional randomized controlled trial designs are often used in this stage. Individuals working at this stage may test bundled strategies, interrogate the “active ingredients” in strategies [46], or test strategies in varied contexts.

An interesting phenomenon occurs in the T3–4 range. Acknowledging the contributions of researchers and practitioners, we see a split whereby researchers continue to serve as the primary actors in one branch of the translational spectrum, while practitioners become the primary actors in another branch of the spectrum. T3, “translation to practice,” includes comparative effectiveness trials and clinical outcome studies and aims to evaluate real-world effectiveness. In D&I, *implementation support practitioners* come into a principal role. Individuals working in this capacity use the results of T0–2 to plan implementation projects, sometimes in the form of quality improvement-type projects. In parallel, T3 *applied implementation researchers* are primarily monitoring or evaluating implementation projects’ real-world effectiveness; this could involve research using pragmatic or naturalistic methods whereby researchers partner with healthcare delivery systems or organizations to better understand real-world implementation or effectiveness outcomes. T4 involves population-level outcomes research and monitoring improvements in morbidity and mortality to impact policy or system change. In D&I, *implementation support practitioners* and *delivery systems* scale EBPs up and out. *Implementation researchers* working at stage T4 define the implementation workforce, develop surveillance systems, and evaluate the effects of evidence-informed implementation on project successes. *Intermediaries*are prime partners in this work. Additional work is needed to establish clear evidence about what is and is not working on a broad scale and in what contexts [42, 47].

Defining the translational spectrum for D&I facilitates the process of identifying a “home base” for individuals involved in D&I science, thereby improving self-concept clarity and making clear how individuals can foray into upstream and downstream segments to better link their research with that of others. In keeping with findings from workplace self-concept clarity literature [48, 49], when we claim our places in the spectrum, we can improve our effectiveness and avoid burnout [50]. Specifically, we can improve our capacity to clearly generate research questions, identify colleagues, and expand the impact of our work.

## How should we do it?

As has been noted by others [21, 51, 52], there is a significant disconnect between individuals working in distinct roles within the field of D&I, particularly between those operating at the two ends of the D&I translational spectrum. By interacting more often and intentionally across the entirety of the D&I process, we as a field could develop significant synergy and produce actionable solutions more quickly to achieve shared goals.

### Asking and answering the right question

Fundamental respect for the work of actors at every level of the implementation spectrum, fostered by regular communication, is essential in resolving our identity crises, achieving our shared goals, and upholding equity within the field [21]. One fundamental way for theoretical implementation scientists to demonstrate respect for implementation practitioners is to ask research questions that implementation practitioners want answered [52]. Implementation practitioners have critical theoretical questions that arise while implementing programs and policies in their specific contexts. For example, implementation practitioners regularly assess organizational readiness for change before altering or implementing a new program or policy (as recommended in the implementation science literature). Yet when the assessments suggest that sites are not ready to implement the intended change, there is little guidance from implementation science about how to best address this issue. A common suggestion is to prioritize “ready” sites [53]. This approach is likely to perpetuate existing inequities or disparities, as “ready” sites are often the sites that are least in need of additional resources and supports, and leaves “non-ready” sites with no plan for reaching a sufficient level of readiness. What strategies can increase readiness? Another example involves the need for a more concrete understanding of the effects of adaptation. While the field might agree adaptation is often important to the scale-up and scale-out of EBPs, many adaptation tools [54, 55] are designed for researchers as opposed to practitioners looking for guidance in understanding if the adaptations they propose will influence the effectiveness of the original EBP. How can D&I measures be made more accessible for implementation practitioners? These are just two examples of many.

### Working with existing implementation efforts

Evaluating existing processes and successes of implementation practitioners can also galvanize efforts, improve impact of D&I, and uphold equity in D&I. Delivery systems are continually implementing “the thing” and have been for years. Connecting with existing implementation efforts and studying the effectiveness of implementation strategies being actively used by delivery systems is critical to supporting the ongoing work of these individuals [2, 21, 56]. In many ways, this can shortcut science more quickly to a clearer understanding of what works when and for whom, and improve the likelihood of establishing sustainable practices and policies that are feasible, acceptable, and appropriate [23, 24]. This approach is also consistent with the principles of community-based participatory research, including respect for lived experience and tailoring interventions to the needs of the community [57, 58].

### Fostering increased communication

Increased communication among actors across the D&I translational spectrum is critical, as previously noted [3, 52, 59]. To again draw from the successes of other fields, the International AIDS Society is a group of over 13,000 members worldwide that “unite(s) scientists, policymakers and activists to galvanize the scientific response, build global solidarity and enhance human dignity for all people living with and affected by HIV” [60]. The International AIDS Society hosts two conferences that rotate annually with a shifting focus between research and practice. Using this model, which has been repeatedly shown to be highly impactful, individuals working at all stages of the HIV implementation science spectrum can engage in, learn from, and contribute to dialogue with others with distinct perspectives and roles in the discipline, thereby improving equity concerning whose voices are centered and uplifted in global agenda-setting efforts. As such, the field of D&I could benefit from an organization akin to the International AIDS Society and agenda-setting practices and conference structures employed by this Society [61–63].

### Developing tools to directly support real-world D&I

Tools that facilitate the translation of D&I into practice are also critical to achieving shared goals [1]. Again, the field of D&I can look to adjacent fields to learn how they have successfully scaled. For example, the Institute for Healthcare Improvement (IHI), whose mission is to improve health and healthcare worldwide, has scaled the use of quality improvement methods. Over 30 years, they have worked in 42 countries and have had over 7 million online course enrollments [64]. Part of IHI’s model has been to develop practical and easy-to-use improvement tools. A critique of implementation science is that existing frameworks are complicated and difficult to use [3, 4]. If the field of D&I learned from the success of IHI and developed tools that help professionals operationalize implementation science in practice, it would support the broader use of D&I to improve outcomes.

### Aligning funding mechanisms and priorities

Funding agencies should increase requirements and supports for community inclusion and implementation throughout the research process. Researchers currently prioritize funding agency policies and expectations, which may not allow enough time for building sustainable community relationships and co-creation of work. A shift in funding agencies’ research calls and approach to awarding research dollars is necessary to build capacity for long-term academic-community partnerships [65–67]. Implementation science-related funding calls from the National Institutes of Health, UK Research and Innovation, the Global Alliance for Chronic Diseases, the South African Medical Research Council, and other funding agencies could more intentionally include requirements for this type of work.

## Conclusion

Key actions are needed for the field of D&I to self-actualize: (1) Uphold everyone’s place at the implementation table while centering the wants and needs of those most directly affected by implementation efforts; (2) Clarify where on the translational spectrum work is being done by whom and where the gaps in both sufficient volume of work and translation of that work lie; and (3) Facilitate regular communication across the spectrum, from theoretical implementation scientists to implementation practitioners and vice versa. Ideally, this work should be done with researchers and practitioners around the globe. If these three tasks are accomplished, we as a field will be able to reverse the tides and bridge the implementation research-to-practice gap, instead of letting it continue to grow.

## Data Availability

Not applicable.

## Abbreviations

D&I: Dissemination and implementation
HIV: Human immunodeficiency virus
AIDS: Acquired immunodeficiency syndrome
